# Control of sand flies with attractive toxic sugar baits (ATSB) and potential impact on non-target organisms in Morocco

**DOI:** 10.1186/s13071-015-0671-2

**Published:** 2015-02-08

**Authors:** Whitney A Qualls, Gunter C Müller, Khalid Khallaayoune, Edita E Revay, Elyes Zhioua, Vasiliy D Kravchenko, Kristopher L Arheart, Rui-De Xue, Yosef Schlein, Axel Hausmann, Daniel L Kline, John C Beier

**Affiliations:** Department of Public Health Sciences, University of Miami Miller School of Medicine, Miami, FL 33136 USA; Department of Microbiology and Molecular Genetics, Kuvin Centre for the Study of Infectious and Tropical Diseases, Faculty of Medicine, Hebrew University, Jerusalem, 91120 Israel; Department of Parasitology, Institut Agronomique et Vétérinaire Hassan II, B.P. 6202 Rabat-Instituts, Morocco; Department of Anatomy and Cell Biology, Bruce Rappaport Faculty of Medicine Technion, 34995 Haifa, Israel; Laboratory of Vector Ecology, Pasteur Institute of Tunis, 13 Place Pasteur BP 74, 1002, Tunis, Tunisia; Department of Zoology, George S. Wise Faculty of Life Sciences, Tel Aviv University, Tel Aviv, 69978 Israel; Anastasia Mosquito Control District, 500 Old Beach Road, St. Augustine, FL, 32080 U.S.A; SNSB-Zoologische Staatssammlung Munchen, Munchhausenstrasse 21, Muunchen, Germany; United States Department of Agriculture-ARS-Center for Medical, Agricultural, and Veterinary Entomology, Gainesville, FL USA

**Keywords:** Integrated vector management, Leishmaniasis, Vector control, Vector-borne diseases, Environmental impact

## Abstract

**Background:**

The persistence and geographical expansion of leishmaniasis is a major public health problem that requires the development of effective integrated vector management strategies for sand fly control. Moreover, these strategies must be economically and environmentally sustainable approaches that can be modified based on the current knowledge of sand fly vector behavior. The efficacy of using attractive toxic sugar baits (ATSB) for sand fly control and the potential impacts of ATSB on non-target organisms in Morocco was investigated.

**Methods:**

Sand fly field experiments were conducted in an agricultural area along the flood plain of the Ourika River. Six study sites (600 m x 600 m); three with “sugar rich” (with cactus hedges bearing countless ripe fruits) environments and three with “sugar poor” (green vegetation only suitable for plant tissue feeding) environments were selected to evaluate ATSB, containing the toxin, dinotefuran. ATSB applications were made either with bait stations or sprayed on non-flowering vegetation. Control sites were established in both sugar rich and sugar poor environments. Field studies evaluating feeding on vegetation treated with attractive (non-toxic) sugar baits (ASB) by non-target arthropods were conducted at both sites with red stained ASB applied to non-flowering vegetation, flowering vegetation, or on bait stations.

**Results:**

At both the sites, a single application of ATSB either applied to vegetation or bait stations significantly reduced densities of both female and male sand flies (*Phlebotomus papatasi* and *P. sergenti*) for the five-week trial period. Sand fly populations were reduced by 82.8% and 76.9% at sugar poor sites having ATSB applied to vegetation or presented as a bait station, respectively and by 78.7% and 83.2%, respectively at sugar rich sites. The potential impact of ATSB on non-targets, if applied on green non-flowering vegetation and bait stations, was low for all non-target groups as only 1% and 0.7% were stained with non-toxic bait respectively when monitored after 24 hours.

**Conclusions:**

The results of this field study demonstrate ATSB effectively controls both female and male sand flies regardless of competing sugar sources. Furthermore, ATSB applied to foliar vegetation and on bait stations has low non-target impact.

## Background

Phlebotomine sand flies are vectors of parasites in the genus *Leishmania* and a number of arthropod-borne viruses primarily in the family Bunyaviridae. Leishmaniasis is recognized as an important but neglected tropical disease with an estimated 350 million people at risk [1]. Both cutaneous and visceral leishmaniasis are vectored by different sand fly species but most cases are of the cutaneous form [2]. In Morocco, both anthroponotic cutaneous leishmaniasis and zoonotic cutaneous leishmaniasis are problematic. Two important species are responsible for the spread of this disease: *Phlebotamus papatasi* and *P. sergenti. Phlebotamus papatasi* is the main vector of zoonotic cutaneous leishmaniasis caused by the etiological agent *Leishmania major* [3]*.* Anthroponotic cutaneous leishmaniasis is caused by the etiological agent *L. tropica* and transmitted primarily by *P. sergenti* [4]. Approximately 3,430 cutaneous leishmaniasis cases have been reported from 2004-2008 with an annual incidence of 9,600 to 15,8000 per 100,000 people [5]. Increasing risk factors related to natural and man-made environmental changes are resulting in the introduction and establishment of new foci of leishmaniasis and other sand fly pathogens [6]. The rapid spread of leishmaniasis and other sand fly vectored pathogens to non-endemic areas require the development of integrated vector management (IVM) strategies for prevention and control of sand flies [7].

Though sand flies pose a major threat to public health, control of sand flies is often difficult because methods mainly rely on interrupting contact between females and humans [8]. For both anthroponotic and zoonotic cutaneous leishmaniasis the only choice is chemical and environmental control. The main chemical control methods have been indoor residual spraying of organochlorines (DDT and dieldrin), organophosphates (malathion), carbamates (propoxur), and synthetic pyrethroids (permethrin and deltamethrin) [9-14]. However, chemical control has not been successful as resistance issues develop and programs face budget cuts that reduce control in areas at risk [15]. A detailed evaluation of a chemical control program on the Tallil Air Base, Iraq identified that air and residual spraying of many different active ingredients had limited impact on sand fly abundance [16]. Environmental control efforts have also failed, as there is a huge knowledge gap in sand fly vector ecology [15]. Economically and environmentally sustainable approaches that can be modified based on the current knowledge of sand fly vector behavior are urgently needed to reduce transmission of sand fly vectored pathogens.

Attractive toxic sugar baits (ATSB) have been successful in controlling mosquitoes, and initial field trials using ATSB for sand fly control have demonstrated significant reductions in sand fly populations [17,18]. The field evaluations have effectively controlled sand flies through ATSB application to patches of vegetation [17] and barrier fences in areas lacking vegetation that could be sprayed [18]. This method has been successful because both male and female sand flies, like other biting flies, require sugar from plants and sometimes honeydew for survival [19-21]. The purpose of this study was to further test the efficacy of ATSB against sand flies while at the same time evaluating the potential impact of the new control method on non-target organisms in the region of Marrakech, Morocco.

## Methods

Sand fly experiments were conducted from early July to late August 2012 in an agricultural area along the flood plain of the Ourika River and nearby hills south of Marrakech, Morocco. The Ourika River, draining from the High Atlas Mountains, creates a fertile valley full of mosaic orchards and fields. The nearby hills are with distance to the plain increasingly arid with sparse dry steppe vegetation. This area is known for its high mosquito [22] and sand fly biting pressure [unpublished data of authors].

### Sand fly experiments

**Figure 1:**
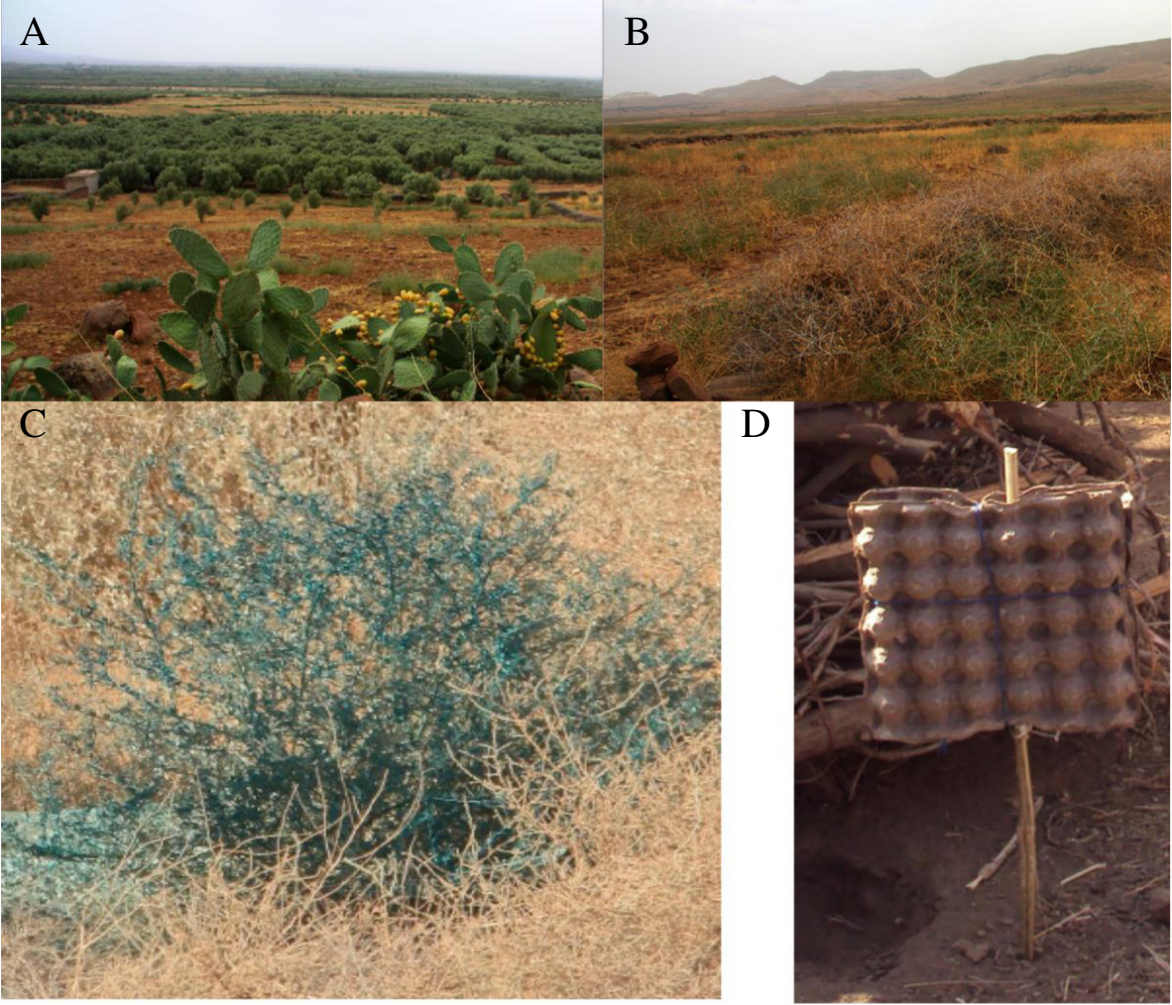
**Depiction of the sugar rich and sugar poor ATSB evaluation sites. A)** sugar rich site with flowering cactus; **B)** sugar poor site with only fallow fields and stone walls, **C)** ATSB dyed vegetation, and **D)** ATSB bait station design using readily available egg cartons.

Six plots of land were selected, each 600m x600m, three with “sugar rich” environments on the flood plain and three in the nearby arid hills with “sugar poor” environments. Sugar rich environments are considered habitats with one or more abundant sugar sources, including flowers, plants with extra floral nectars, fruits or honeydew soiled non-flowering vegetation. In the present study the dominant sugar source was cactus fruits (*Opuntia ficus-indica*) (Figure 1A). Sugar poor environments are habitats without the above described sugar sources (Figure 1B) only with green plants suitable for plant tissue feeding. Fallow fields, mainly with bare soil or with patches of dry annual vegetation including semi-shrubs, and long stone walls dominated both sites. At the time of the experiments there was little flowering and no honeydew soiled vegetation found. Each of the three plots at the sugar rich site were surrounded by cactus hedges, full with ripe fruit, and plenty of partially rotting fruits on the ground.

### Mixture and application of baits

Attractive sugar baits (ASB) were prepared from industrial grade attractive sugar bait and a preservative BaitStab® concentrate (Westham Ltd, Tel Aviv, Israel) by diluting concentrate 1:3 in regular tap water. ATSB was prepared by adding dinotefuran (Safari™ 20 SG, Valent USA Corporation, Walnut Creek, CA) at 100mg/L to the ASB concentrate. Such prepared baits are typically invisible after once applied on vegetation. For experimental reasons we added red food dye (1:200 Azorubine food dye, Stern, Natanya, Israel) to stain the baits. The food dye stains the guts of insects that fed on the bait for at least 24 h. The percentage of stained insects after the first day of ASB application can therefore be seen as a potential maximal daily feeding/ killing rate [23].

At both the sugar rich and sugar poor sites one site was treated with either ATSB applied to vegetation or applied to bait stations (Figure 1C). ASB was sprayed at both sugar rich and sugar poor sites for non-target studies (described below). Control sites were also located in both the sugar rich and sugar poor site. The ATSB and ASB formulation was sprayed with a backpack sprayer on sugar rich and sugar poor vegetation while moving the nozzle up and downwards to cover both the under and upper side of the foliage (Solo® Backpack Sprayer, Newport News, VA).

The ATSB formulation was applied to bait stations first by dipping the bait station into the concentrate. The bait stations were allowed to dry for 2 hrs and then dipped a second time in the ATSB concentrate. As bait stations we used locally acquired egg cartons mounted on wooden poles with the bottom of the bait station around 30 cm above the ground (Figure 1D). The bait stations were placed out every 50 meters for a total of 12 bait stations per site.

Sand fly populations were monitored for the ATSB/ASB experiments with 6 UV-tray traps (total of 36 traps) [24] placed in each sugar rich and sugar poor site four times a week, one week prior to ATSB/ASB application and twice a week after application for five weeks. From each of the six sites, a random sample of 50 sand flies (300) was identified to species in order to determine the sand fly species composition during both the pre-treatment and post-treatment periods.

### Non-target field experiments

Field experiments with non-targets were conducted with the assumption that all insects feeding directly on ATSB treated foliage would eventually die. Before death they would exhibit behavioral changes, which would make it difficult to collect these insects affected by ATSB in experimental areas in amounts comparable to untreated control sites. It was decided to use non-toxic but color-stained ASB to explore attraction and feeding of both target and non-target insects as the best method to obtain representative results from the field.

Field studies evaluating feeding on vegetation treated with ASB by non-target insects were conducted by dissecting and examining guts for food dye under a dissecting microscope. The insect orders included: Hymenoptera **(**with focus on Aculeata including honey bee (*Apis mellifera*), wild bees and wasps), Lepidoptera **(**with focus on adults of Erebidae, Noctuidae, Geometridae, Pyralidae), Coleoptera **(**with focus on Tenebrionidae, Scarabaeidae, Cerambycidae, Chrysomelidae), Diptera (Brachycera only), Orthoptera (Caelifera and Ensifera), and Neuroptera (Chrysopidae and Myrmeleontidae). The experiments were designed under the assumption that, under natural conditions, at least half of the individuals will remain at the same site after 24 hours [25] and thus will be available for monitoring. The focus groups of non-target insects were chosen to include high portions of species with low dispersal and high portions of nectar- and leave-feeders.

Non-target field studies were conducted at the sugar rich and sugar poor sites, one plot each, by spraying approximately 10% of the local vegetation with red stained ASB, the other by applying red stained ASB on bait stations. One plot at each site was left untreated as a control. The vegetation was treated in 0.5 mx0.5m spots or 0.5m strips in intervals depending on the vegetation cover at the sites while the bait stations were arranged in a grid pattern (1 station/400m^2^) [18]. In the absence of specific EPA guidelines, we specifically designed experiments coming as close as possible to use of the product under field conditions. Testing was performed under field trail conditions with ATSB foliar spray application on non-flowering plants per product label instructions [26-28].

Non-target insects were monitored one day/night after ASB application at the treated site with 50 yellow plates (yellow disposable plastic plates 25 cm diameter filled with water and a drop of triton-x as detergent), four Malaise traps (2 and 6 m; Model 2875D, BioQuip, Rancho Dominguez, CA), two large UV-light traps (generator powered 250 ML light bulb mounted in front a white 2 × 5 m white linen sheet), six UV-tray traps [24], 50 pitfall traps (500ml plastic cups buried to the rim in the ground, baited with 10ml vinegar), sweep-nets (BioQuip, Rancho Dominguez, CA) (two collectors), and entomological hand nets (BioQuip, Rancho Dominguez, CA) (two collectors) [29,30]. Collected insects were immediately killed in collecting jars (BioQuip, Rancho Dominguez, CA) with Ethyl acetate and stored in a freezer (-20°C) before processing.

Because of the large number of non-targets that were collected, aliquots from each collecting method were used to determine the percentage of stained insects. Again due to the volume of the collections morpho-species, (species that are distinct based on morphological characteristics), were identified instead of identifying each specimen to species level.

### Data analysis

**Figure 2:**
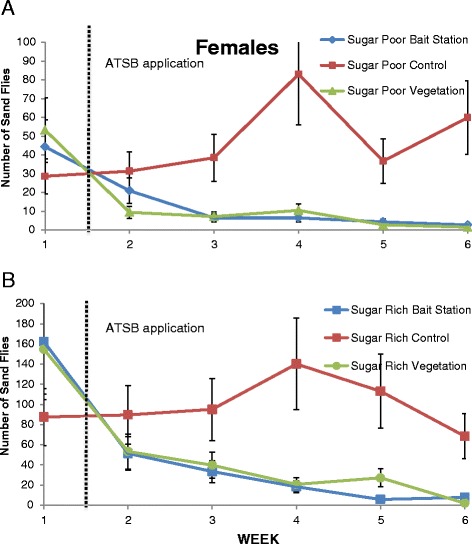
**Number of female sand flies pre- and post- ATSB application collected at. A)** sugar poor sites and **B)** sugar rich sites.

Counts of male and female sandflies trapped over time was analyzed by a generalized linear model for a Poisson distribution. The model included effects for group (sugar rich control, sugar rich bait, sugar rich vegetation, sugar poor control, sugar poor bait, sugar poor vegetation), day (1-6), and the interaction of group and day. A negative binomial link was used to accommodate the overdispersion of the sandfly counts. Planned comparisons were made among the control, bait, and vegetation measures within the sugar rich and poor conditions at each time. Results are shown as plots of means and standard errors in Figures 1 and 2. Percent change was analyzed with a linear mixed model. The fixed effects were the same six groups discussed above, day (2-6 and one week), and the interaction of group and day. A random effect of trap nested within group was included, and a heterogeneous autocorrelated covariance matrix was used to represent the correlated data structure. Each mean percent change was tested to determine if it was significantly different from zero. The counts of trapped and stained insects of each species had a Poisson distribution. Therefore, we used a generalized linear model to compare the number of stained insects of each species. We used the total number of each species as an offset to produce proportions of stained insects for each species. Since there was marked over-dispersion, a negative binomial link was used. The results were reported as the percent and standard error of stained insects for each species. The 0.05 significance level was used to determine statistical significance. SAS 9.3 (SAS Institute, Inc., Cary, NC) was used for all analyses.

## Results

### Sand fly field experiments

From a random sample of 50 sand flies from each of the six sites (300 flies) before the experiments commenced, 98% (294/300) were *P. papatasi* and 2% (6/300) were *P. sergenti.* During the ATSB bait station evaluation, 94% (282/300) of the sand flies sampled were *P. papatasi* and the remaining specimens were *P. sergenti*(8/300). The species composition was similar to another study conducted in urban areas of Marrakech, Morocco [31]. There was no significant difference in the species assemblage between the six sites nor before or after the treatment (P >0.05).

At both the sugar-poor and sugar-rich site, a single application of ATSB either applied to vegetation or bait stations significantly reduced densities of both female (F=25.7, df=5,30, P<0.001) and male (F=28.3, df=5,30, P<0.001) sand flies. Sand fly populations were reduced at the end of the 4-week field trial by 82.8% and 76.9% at sugar-poor sites having ATSB applied to vegetation or presented as bait stations, respectively. At the sugar-rich sites sand fly populations were reduced at the end of the 4-week field trial at both ATSB vegetation and bait station site, by 78.7% and 83.2%, respectively.

**Figure 3:**
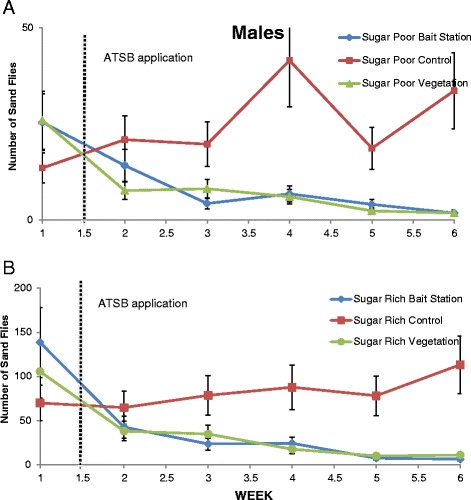
**Number of male sand flies pre- and post- ATSB application collected at. A)** sugar poor sites and **B)** sugar rich sites.

Table 1 demonstrates the average decrease or increase in female and male sand fly populations at sugar poor and sugar rich sites following ATSB applied to vegetation, bait stations, or untreated areas. Each week post-ATSB application to green vegetation at either sugar poor or sugar rich site showed a significant reduction in female (Figure 2) and male (Figure 3) sand fly populations. There was a significant reduction in female and male sand fly populations each week post-ATSB presented as bait stations in both sugar poor (Figures 2A and 3A) and sugar rich sites (Figures 2B and 3B). There was a natural increase in control female and male populations that did not receive an ATSB treatment. In the sugar poor control site on week 1 post-treatment application there was a significant increase in female sand fly populations compared to pre-treatment numbers (P=0.021). However, for all other weeks regardless of site, sugar poor or sugar rich, there was no significant difference recorded in mean female sand fly populations compared to the pre-treatment. In the sugar poor control site on week 1 and 5 post-treatment application there was a significant increase in male sand fly populations compared to pre-treatment numbers (P=0.21). However, for all other weeks regardless of site, sugar poor or sugar rich, there was no significant difference recorded in mean male sand fly populations compared to the pre-treatment.

**Table 1:** **Pre-treatment and Post-treatment female and male sand fly averages ± SE at sugar poor and sugar rich sites following ATSB applied to vegetation, bait stations, or untreated areas**

**Sites**	**Female**		**Male**	
	**Pre-treatment**	**Post-treatment**	**Pre-treatment**	**Post-treatment**
Sugar-poor				
Green vegetation	53.2 ± 17.2	1.5 ± 0.7	25.9 ± 7.5	1.8 ± 0.8
Bait station	44.2 ± 14.3	2.7 ± 1.1	25.4 ± 7.4	1.8 ± 0.8
Untreated	28.6 ± 9.3	59.8 ± 19.5	13.6 ± 3.9	33.7 ± 10.0
Sugar-rich				
Green vegetation	154.8 ± 49.9	2.2 ± 0.9	105.6 ± 30.2	11.0 ± 3.4
Bait station	162.2 ± 52.4	7.8 ± 2.7	138.6 ± 42.4	6.3 ± 2.1
Untreated	87.5 ± 28.3	113.1 ± 36.5	69.9 ± 20.1	113.3 ± 32.7

### Non-target experiment

The potential impact of ATSB on non-targets, if applied on green non-flowering vegetation and bait stations, was low for all non-target groups as only 1.0% and 0.7% were stained respectively. However, ASB application on flowering vegetation suggested a high impact on most non-target groups. On average 8.4% of the (recaptured) investigated insect groups fed on stained ASB on flowering vegetation, especially some groups of Coleoptera, Lepidoptera, and Hymenoptera would have been affected at unsustainable rates (Table 2). The potential impact on sand flies was high regardless of the application method and would have been sufficient for control. The percent stained for the sand flies was 29%, 36.2%, and 61%, for ASB applied to bait stations, non-flowering vegetation, and flowering vegetation, respectively (Table 2).

**Table 2:** **Percent stained (Mean) and SE of stained insects in each order compared to sand flies (control group) for each ASB application type**

	**Bait station**			**Non-flowering vegetation**			**Flowering vegetation**		
**Target/Non targets**	**Mean**	**SE**	**P-value** ^**1**^	**Mean**	**SE**	**P-value** ^**1**^	**Mean**	**SE**	**P-value** ^**1**^
sand flies	29.25	27.91		36.20	24.23		61.00	71.77	
Coleoptera	0.16	0.13	0.001	0.49	0.29	<0.001	6.46	3.22	0.097
Diptera*	1.37	1.32	0.037	2.88	1.95	0.017	18.70	21.89	0.486
Hemiptera	0.09	0.12	0.002	0.51	0.33	<0.001	2.25	1.94	0.037
Hymenoptera	0.66	0.38	0.003	0.67	0.29	<0.001	14.74	9.97	0.310
Lepidoptera	0.76	0.34	0.003	0.93	0.32	<0.001	3.40	1.65	0.037
Neuroptera	0.15	0.20	0.005	0.31	0.26	<0.001	2.53	1.84	0.034
Orthoptera	0.23	0.27	0.006	1.46	0.84	0.002	2.75	1.95	0.038

^1^Comparison of insects of all orders to the control group (sand flies).

*without sand flies.

## Discussion

At both the sugar rich and sugar poor sites, the ATSB-dinotefuran application to vegetation or bait stations was effective compared to the control sites in reducing sand fly populations for five weeks post-application. Both application strategies worked well in sugar rich and sugar poor sites but the observed decline in mosquito populations took longer in the sugar rich sites. The current findings of this study support preliminary studies evaluating ATSB for control of sand flies [17,18]. In the previous studies *P.papatasi* populations were reduced by 95% after application to natural habitats in Jordan Valley [17]. In the same study *Phlebotomus syriacus, P. sergenti, Phlebotomus tobbi* and *P. papatasi* were controlled with repetitive treatments of ATSB in an urban setting over a complete sand fly season. Müller and Schlein [18] demonstrated a significant reduction in *P. papatasi* after ATSB application to vegetation and barrier fences and suggested that at least in arid areas where attractive flowering plants are scarce ATSB will continue to be successful for sand fly control. However, even in the presences of other competing sugar sources, this study demonstrates that ATSB can be successful in reducing sand fly populations. This success highlights the extremely attractive nature of the attractant in the ATSB mixture. This attribute of ATSB suggests that this method can be effective in diverse ecological settings, possibly even in tropical environments where sugar source competition is highest [32-35].

ATSB with the toxin dinotefuran has successful controlled *Culex* and *Aedes* spp. in similar field sites in Morocco [22]. However, this if the first report of the use of dinotefuran for control of sand flies. Dinotefuran is a neonicotinoid that acts as agonist on the nicotinic acetylcholine receptor [36]. The addition of dinotefuran can mitigate resistance issues in sand fly IVM programs since there is no associated pyrethroid or carbamate cross-resistance [37]. In addition, ATSB has the ability to be manufactured with many different active ingredients that act as gut toxins. These toxins include Environmental Protection Agency ingredients that are consider low risk because of their low mammalian toxicity. This facet of ATSB can circumvent major issues faced by sand fly control programs that have relied on the use of contact insecticides. Failures for sand fly control following application of residual insecticides have been associated with a non-lethal dose because of limited contact with the insecticide [38]. With ATSB the insecticide is ingested and thus the sand fly exposed to the active ingredient for a long time.

In this study there were no obvious negative impacts of the ASB method observed on non-target arthropod populations in the field evaluations in Morocco. The current study corroborates previous studies where ATSB applications had very little impact on non-target populations specifically pollinators of flowers [22,32,39]. Khallaayoune et al. [19] conducted similar experiments in Morocco on non-target populations and found that when ATSB was applied to non-flowering vegetation <1% of non-target arthropods were found to have fed on the dyed ASB solution. Revay et al. and Qualls et al. [32,39] both found in field trials conducted in Florida, USA, that when ASB was applied to non-flowering vegetation the impact on non-target arthropods was very low for all orders as <1% of the(0.6 and 0.9%, respectively) individual insects were stained with the dye from the sugar solutions. Thus, as demonstrated in this study, when ASB was applied on non-flowering vegetation or to bait stations, non-target insect populations were not attracted to the baits and did not feed on them. However, when ASB was applied to flowering vegetation the staining rate of non-targets was considerably higher suggesting that some non-target populations in the case of a toxic bait would have been unable to recover [22,32,39]. Most likely, the ASB-treated green vegetation does not provide a visual attractive target for pollinators providing an explanation for our findings. In order to stand out from the predominant green colors of leaves and stems, plants have flowers and fruits that vary in color and shape. Both create optical signals that are used to attract insect pollinators [40]. As a result, ATSB applications, as long as they are applied to green, non-flowering vegetation or in bait stations would have little attractancy to the adults of most non-target populations and avoid any potential unacceptable negative impact. This study continues to provide essential non-target data that is needed for the development of clear guidelines for appropriate use of ATSB control method for guiding the environmentally friendly but effective treatment. Low non-target impacts are also an important concept to consider when implementing IVM programs. So far, the non-target experiments were restricted to adult insects. Further research is needed to investigate also the effects of ATSB foliage treatment on immature stages, which should be monitored after defined intervals by combined, semi-quantitative methods such as fogging, branch-beating, and net-sweeping.

While non-target adult arthropods were not attracted to nor feeding on the ASB foliar application, sand flies had a high level of daily staining, > 40%, indicating ingestion of the bait at levels likely sufficient for control. Sand flies appear to be guided more by scent than optical targets when sugar seeking similar to host-seeking which is only to a minor part influenced by optical cues like color and shape of the object [41]. Müller et al. [42] demonstrated effective control in storm drain systems by comparing the number of mosquitoes feeding on the ASB solution to the numbers after an ATSB application. Qualls et al. [43] demonstrated a high level of staining from feeding on ASB (90%) by mosquitoes emerging from cisterns and wells. Other studies incorporating a dyed ASB control have demonstrated in most cases > 50% daily staining rate while achieving at least that percentage of control in the ATSB treatment sites [44-46].

The single application of ATSB demonstrated effective control for sand fly populations during the one-month post-treatment evaluation period. The field trials were completed under a time restraint and it is possible that had the sand fly populations continued to be monitored that the residual effect in the Mediterranean rainless condition could have far exceeded five weeks. In trials in Israel and Mali the next generation of bait stations containing the Baitstab® (developed by Westham Innovations, LTD, Israel) covered with a biofilm were highly attractive and killing mosquitoes up to 6 months (unpublished data). The development of an ATSB with a residual of six months or more provides an operational strategy that is economically feasible for the development of sand fly IVM programs. Leishmaniasis and other sand fly diseases are usually associated with socio-economically poor countries [2,6]. Thus, the development of a long lasting bait station or even ATSB reapplications to vegetation located near identified sand fly developing sites could result in economically and environmentally friendly strategies for sand flies.

Further studies using ATSB for control of sand flies in diverse eco-zone and eco-systems are needed to develop this strategy for incorporation into IVM programs for sand fly control. As a rule, IVM programs should be based on behavioral and ecological knowledge of the target organism to design strategies for optimal results. Currently, there are limited methods for effective sand fly control mainly due to the fact that there are key gaps in sand fly ecology, which limits IVM strategies. However, the success of the ATSB method in both sugar rich and sugar poor environments provides evidence that sand flies: 1) are frequently feeding on sugar, enhancing our understanding of key aspect of sand fly ecology and 2) that ATSB is highly attractive and competitive with natural sugar sources so much that sand fly control can be achieved.

## Conclusion

The results of this study demonstrate that ATSB has real operational potential to be used in IVM programs for sand fly control in countries experiencing the burden of leishmaniasis and other important sand fly vectored pathogens. ATSB is especially promising because this method is environmentally friendly, economically feasible, and sustainable. Importantly, this study identifies that targeting specific behaviors of sand flies results in successful control and thus importance should be placed on understanding sand fly ecology for the development and implementation of control methods.
